# Saccharin

**Published:** 1890-03-01

**Authors:** 


### THERAPEUTICS.

### SACCHARIN.

Many different opinions have been expressed with reference to saccharin. The most recent is that of Stift. To ascertain the action of saccharin on digestive ferments experiments were made with meat, egg, albumen, casein, pea-meal, &c., with and without the addition of saccharin. The presence of saccharin delayed the solution of albuminoids, which was not complete until twelve hours after contact with the gastric juice. Saccharin must therefore be regarded as a substance which interferes with digestion, and is therefore injurious. Further, saccharin is not capable of being itself digested, but passes unchanged through the organism. Loss of appetite and slight purgative action were observed as the effect of saccharin on some persons. If this be correct it cannot be beneficial for diabetic patients, for whom it has been recommended

